# Axon–axon interactions determine modality-specific wiring and subcellular synaptic specificity in a somatosensory circuit

**DOI:** 10.1242/dev.199832

**Published:** 2023-03-15

**Authors:** Samantha E. Galindo, Abby J. Wood, Patricia C. Cooney, Luke A. Hammond, Wesley B. Grueber

**Affiliations:** ^1^ Department of Genetics and Development, Vagelos College of Physicians and Surgeons, Columbia University, New York, NY 10032, USA; ^2^ Mortimer B. Zuckerman Mind Brain Behavior Institute, Columbia University, New York, NY 10027, USA; ^3^ Department of Neuroscience, Mortimer B. Zuckerman Mind Brain Behavior Institute, Vagelos College of Physicians and Surgeons, Columbia University, New York, NY 10027, USA; ^4^ Department of Physiology and Cellular Biophysics, Vagelos College of Physicians and Surgeons, Columbia University, New York, NY 10032, USA

**Keywords:** Axon targeting, *Drosophila*, Neural circuit, Somatosensory neurons, Synaptic specificity

## Abstract

Synaptic connections between neurons are often formed in precise subcellular regions of dendritic arbors with implications for information processing within neurons. Cell–cell interactions are widely important for circuit wiring; however, their role in subcellular specificity is not well understood. We studied the role of axon–axon interactions in precise targeting and subcellular wiring of *Drosophila* somatosensory circuitry. Axons of nociceptive and gentle touch neurons terminate in adjacent, non-overlapping layers in the central nervous system (CNS). Nociceptor and touch receptor axons synapse onto distinct dendritic regions of a second-order interneuron, the dendrites of which span these layers, forming touch-specific and nociceptive-specific connectivity. We found that nociceptor ablation elicited extension of touch receptor axons and presynapses into the nociceptor recipient region, supporting a role for axon–axon interactions in somatosensory wiring. Conversely, touch receptor ablation did not lead to expansion of nociceptor axons, consistent with unidirectional axon–axon interactions. Live imaging provided evidence for sequential arborization of nociceptive and touch neuron axons in the CNS. We propose that axon–axon interactions and modality-specific timing of axon targeting play key roles in subcellular connection specificity of somatosensory circuitry.

## Teaser

**Highlighted Article:** Axons of nociceptive neurons impact the modality-specific wiring and subcellular synaptic specificity of touch receptor axons in a *Drosophila* somatosensory circuit.

## INTRODUCTION

The specificity of synaptic connections between neurons underlies information transfer and circuit function throughout the nervous system. Mechanisms that promote synaptic specificity must restrict pairing to correct partners and exclude inappropriate connections, all within a highly complex cellular environment. This monumental task is the culmination of several incremental, well-orchestrated steps, including cell migration, neuronal polarization, axon and dendrite growth, and target recognition, carried out repeatedly throughout the developing nervous system. Specificity often involves synapse formation at particular subcellular locations on a postsynaptic target (Sanes and Yamagata, 2009; Yogev and Shen, 2014). Thus, locating an appropriate synaptic partner is one step in synaptic specificity, and connectivity in a particular spatial pattern upon that partner is another. Moreover, as arbors scale to maintain relative proportions and target coverage during animal growth, there could conceivably be additional requirements for maintenance of connectivity patterns throughout development. An understanding of the cellular and molecular mechanisms that underlie each step of synaptic specificity, in particular the later steps in subcellular specificity, remains a major unmet goal in neuroscience.

Subcellular specificity of synaptic connections is a central feature of neuronal circuits that compartmentalizes information transfer and affects how signals are integrated by postsynaptic targets (Kastellakis et al., 2015; Stuart and Spruston, 2015; Vlasits et al., 2016). Many factors can contribute to subcellular synaptic specificity including target-derived and target-independent mechanisms. Differential expression of membrane molecules on postsynaptic targets can instruct axon placement (Ango et al., 2004; Nishimura-Akiyoshi et al., 2007). Guidance molecules can recruit axons to different regions of a postsynaptic dendrite (Sales et al., 2019; Suto et al., 2007). Axon–axon interactions also play important roles in axon targeting (Imai and Sakano, 2011; Wang and Marquardt, 2013). However, the extent to which axon–axon interactions influence subcellular synaptic specificity is poorly understood. Here, we explore how axon–axon interactions influence axon targeting and subcellular synaptic specificity in a well-characterized somatosensory circuit in *Drosophila*.

We recently identified precise targeting and potential subcellular synaptic specificity in the *Drosophila* somatosensory system. Two distinct types of somatosensory neuron, class III (cIII) touch sensing and class IV (cIV) nociceptive neurons, target axons to adjacent areas in the neuropil of the larval ventral nerve cord (VNC) (Burgos et al., 2018; Grueber et al., 2002; Grueber et al., 2007). The close juxtaposition of cIV and cIII terminals suggested that axon–axon interactions could mediate bundling of like-type axons and/or segregation of non-like axons. Both axon cohorts synapse on down-and-back (DnB) nociceptive interneurons, but appear to do so on distinct regions of the DnB dendritic arbor (Burgos et al., 2018), with cIV axons terminating on the medial region of the DnB dendrite and cIII axons terminating on an adjacent lateral region (Burgos et al., 2018). Here, we explore the role of axon–axon interactions in the targeting of clV and cIII axons and the impacts of these interactions on subcellular connection specificity.

## RESULTS

### cIII and cIV axon terminals occupy non-overlapping zones in the central nervous system (CNS)

**Fig. 1.**
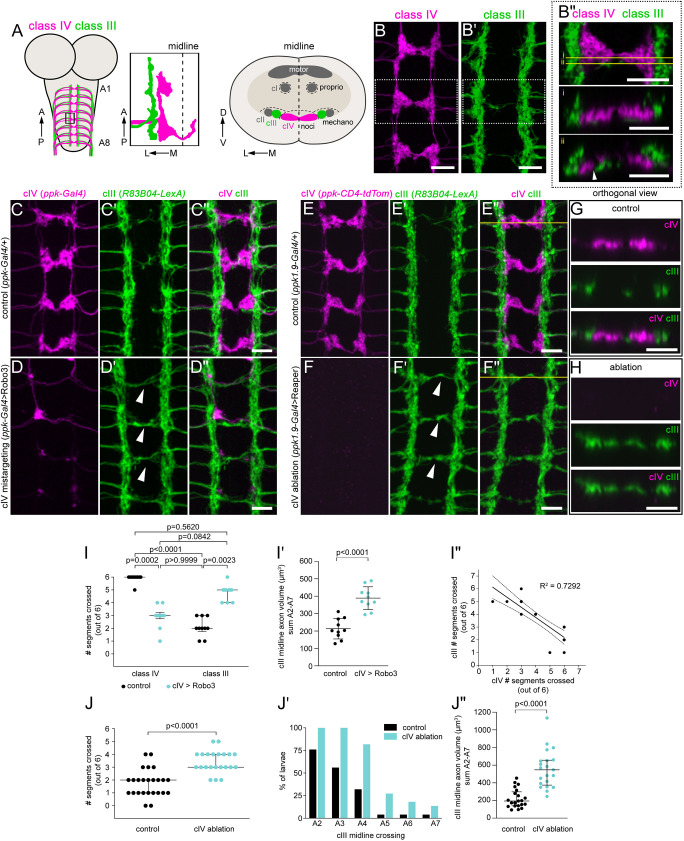
**cIV nociceptor axons are required for lateral restriction of cIII touch receptor axons.** (A) Illustration of class IV (cIV) and class III (cIII) axons in larval central nervous system (CNS). (Left) Axon terminals in abdominal segments (A1-A8) in the ventral nerve cord (VNC) project along the anterior (A)–posterior (P) axis. (Center) More detailed illustration of the boxed area on the left, indicating the precise layering of cIV (magenta) and cIII (green) axons. Note that cIV axons cross the midline. (Right) A schematic of a transverse view of the VNC shows the ventral location of somatosensory axons examined in this study relative to the locations of proprioceptor axons and motor neuropil. cI, class I; cII, class II; D, dorsal; L, lateral; M, medial; mechano, mechanoreceptive; noci, nociceptive; proprio, proprioceptive; V, ventral. (B,B′) cIV and cIII axons (segments A3-A5) co-labeled by *ppk-CD4-tdTomato* (B; magenta) and *R83B04-LexA* (B′; green), respectively. (B″) (Top) Single-plane view of the boxed region in B and B′ (*z*=0.5 µm). (Center, bottom) Orthogonal views from the positions of the yellow lines in the top image. Arrowhead points to homotypic bundling and heterotypic segregation of cIV and cIII axons at midline. (C-D″) cIV and cIII axons (segments A2-A5) in control (C-C″) and cIV mistargeted (D-D″) VNC. Arrowheads in D′ indicate cIII axons that span the midline. (E-F″) cIV and cIII axons from segments A2-A5 in control (E-E″) and cIV-ablated (F-F″) VNC. Arrowheads in F′ indicate cIII axons that span the midline. (G) Orthogonal views of E-E″ (top to bottom) at the position of the yellow line in E″. (H) Orthogonal views of F-F″ (top to bottom) at the position of the yellow line F″. (I) Quantifications of cIV and cIII midline crossing in control and cIV-mistargeted larvae. Control cIV versus Robo3 cIV, *P*=0.0002; control cIV versus control cIII, *P*<0.0001; control cIV versus Robo3 cIII, *P*=0.5620; Robo3 cIV versus control cIII, *P*>0.9999; Robo3 cIV versus Robo3 cIII, *P*=0.0842; control cIII versus Robo3 cIII, *P*=0.0023; Kruskal–Wallis with Dunn's multiple comparison test. *n*=10 larvae for both groups. Each dot is one larva, and medians±interquartile ranges are shown. (I′) Quantification of cIII axon volume in the midline region (segments A2-A7) in control and cIV-mistargeted larvae. *P*<0.0001 by two-tailed unpaired *t*-test. *n* values are the same as in I. Each dot is one larva, and means±s.d. are shown. (I″) Linear regression of the number of segments with cIII axons spanning the midline versus the number of segments with cIV axons spanning midline. *R*^2^=0.7292. Dotted lines represent 95% c.i. *n* values are the same as in I and I′. (J,J′) Quantification of cIII midline crossing in control and cIV-ablated larvae. *P*<0.0001 by Mann–Whitney test. Control, *n*=25 larvae; ablation, *n*=22 larvae. Each dot in J is one larva, and medians±interquartile ranges are shown. J′ shows percentage of larvae with cIII axons spanning the midline. (J″) Quantification of cIII axon volume in the midline region (segments A2-A7) in control and cIV-ablated larvae. *P*<0.0001 by Mann–Whitney test. *n*=21 larvae for both groups. Each dot is one larva, and medians±interquartile ranges are shown. All samples shown in images were labeled using immunohistochemistry. Scale bars: 10 µm.

Prior results showed that axons of different classes of dendritic arborization (da) neurons terminate in adjacent regions of the ventral neuropil (Fig. 1A), leading to a modality-specific medial-to-lateral arrangement of cIV (most medial), cIII and class II (cII; most lateral) somatosensory neurons (Grueber et al., 2007). However, these conclusions were based on rare instances in which two cells from the same segment were sparsely labeled using FLP-out or mosaic analysis with a repressible cell marker (MARCM) approaches (Grueber et al., 2007). Segregation of cIII and cIV axons was observed with co-labeling of cohorts of the different classes as well (Burgos et al., 2018). To confirm these findings and examine the mechanisms of segregation, we examined axon terminal organization using cIV- and cIII-specific markers. In a screen of Janelia Gal4 lines (Jenett et al., 2012), we found that *R83B04-Gal4* drove reporter expression in cIII neurons in third-instar larvae and some cells in the brain lobes. No other cells in the VNC or peripheral nervous system were robustly labeled; thus, we could unambiguously assign VNC axons to cIII neurons (Fig. S1A). We generated *R83B04*-*LexA*, which likewise labeled cIII neurons and occasionally the dorsal multidendritic (dmd1) proprioceptor (Fig. S1B). dmd1 projects an axon dorsally in the neuropil, which could easily be distinguished from the ventrally situated axons of cII, cIII and cIV neurons (Corty et al., 2016). We co-labeled cIII and cIV axons by combining *R83B04-LexA>LexAop-myr-GFP* with *ppk-CD4-tdTomato* (Han et al., 2011) (Fig. 1B-B″). As reported previously, cIV axons collectively form a ladder-like arrangement and cross the midline (Burgos et al., 2018; Grueber et al., 2003, 2007). cIII axons formed parallel longitudinal tracts lateral to the cIV longitudinal connectives and largely avoided the midline. A few cIII axons extended toward or across the midline, but these remained segregated from the cIV midline axon bundle (Fig. 1B″). Notably, cIII and cIV axon tract boundaries showed striking shape complementarity (Fig. 1B″), indicating precise modality-specific segregation of somatosensory axons.

### cIV axons are required for lateral restriction of cIII axons

Given the tight apposition of cIII and cIV axon terminal cohorts, we hypothesized that cIV axons impact the terminal locations of cIII axons. We tested this first by inducing mistargeting of cIV axons and assessing whether cIII axons showed cell non-autonomous changes in targeting. Overexpression of Robo3 in all da neurons causes axon lateralization and loss of cIV midline crossing (Grueber et al., 2007). Likewise, overexpression of Robo3 selectively in cIV neurons decreased midline crossing of axon terminals, as reflected by the lack of commissures (Fig. 1C,D,I). Compared with control larvae, larvae with disrupted cIV axon terminals had a significant increase in midline crossing by cIII axon terminals, as measured by the number of segments with cIII axons spanning the midline (Fig. 1C′-D″,I) as well as total volume of cIII axons in the midline region (Fig. 1I′). Linear regression revealed a strong correlation between cIII axons spanning the midline and the lack of cIV axons spanning the midline (Fig. 1I″). These data suggest that the presence of cIV axons prevents cIII axons from approaching the midline.

One caveat of Robo3 overexpression is that high-level presentation of this axon guidance receptor on cIV axons could conceivably alter the local cues seen by cIII axons. We therefore assessed the requirement for cIV axons in restricting cIII axons from the midline by genetically ablating cIV neurons with *UAS-reaper* (McNabb et al., 1997). We labeled cIV neurons using *ppk-CD4-tdTomato* and labeled cIII neurons using *R83B04-LexA*>*LexAop-myr-GFP*. cIV ablation led to a significant increase in cIII midline crossing compared with that in control larvae (Fig. 1E-F″,J-J″). cIII axons extended along the ventromedial neuropil and, like cIV terminals, crossed the midline ventrally (Fig. 1G,H). These results indicate that cIV neurons restrict bundles of cIII axons to longitudinal tracts and prevent extensive midline projections.

### cIV axons are required to maintain positioning of cIII axons

**Fig. 2.**
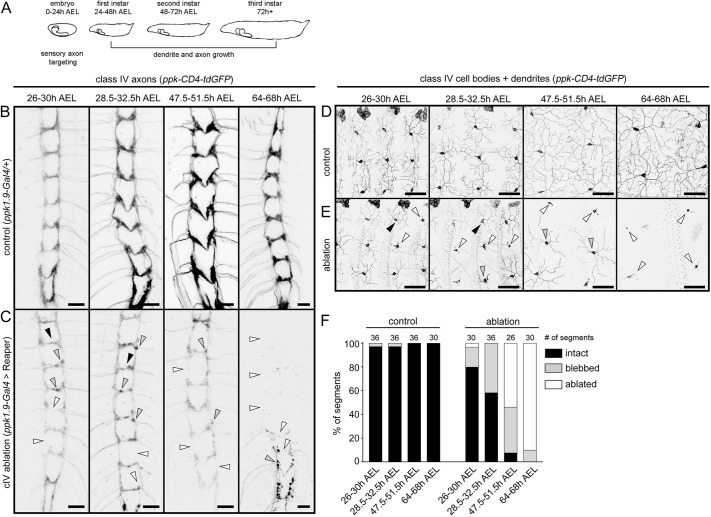
**Time course of cIV genetic ablation.** (A) Timeline of embryonic and larval peripheral nervous system development showing major events in somatosensory neuron development. AEL, after egg laying. (B,C) Axon scaffold in control (B) and cIV-ablated (C) larvae imaged at various stages of development. Black arrowheads indicate intact segments; gray arrowheads indicate segments with blebbing; white arrowheads indicate segments with missing axons. (D,E) cIV cell bodies and dendrites in control (D) and cIV-ablated (E) larvae. Ages correspond to B and C. Black, gray and white arrowheads indicate intact, partially ablated and fully ablated dendrites, respectively. (F) Quantification of cIV axon loss. CNS segments (A2-A6) were analyzed individually and pooled by larval age and genotype. Numbers of CNS segments analyzed per group are displayed above bars. All images were acquired using live imaging to detect *ppk-CD4-tdGFP*. Scale bars: 10 µm (B,C); 50 µm (D,E).

Although axon sorting occurs during embryonic development (Grueber et al., 2007), axons continue to grow throughout larval development (Gerhard et al., 2017). Thus, cIV axons could be required during initial targeting to position cIII axons, continuously to maintain the location of cIII axons, or both. To determine when cIV axons impact cIII axon targeting, we monitored the timing of cIV ablation induced by *UAS-reaper*. We visualized cIV axons and dendrites in first- and second-instar larvae using *ppk-CD4-tdGFP* (Han et al., 2011). In first-instar larvae [26-32.5 h after egg laying (AEL); Fig. 2A] expressing *UAS-reaper* in cIV neurons, we observed axon blebbing, which has been described in axons of cIV neurons following induction of apoptosis (Smart et al., 2017). We also observed a few segments of the CNS in which axons were missing (Fig. 2B,C,F). Loss of axons was accompanied by dendrite loss in the periphery (Fig. 2D,E). By the second-instar stage (47.5-68 h AEL), cIV axon and dendrite loss became more extensive and only a few axons remained by 68 h AEL. By contrast, axon loss was not observed in age-matched controls. As most cell death occurred after axon targeting, these results suggest that cIV axons are required to maintain cIII axon positioning. Given the late timing of cIV ablation, we cannot rule out an earlier role for axon interactions during initial targeting.

### cIV axon targeting is not altered after cIII neuron ablation or upon manipulation of a postsynaptic target

**Fig. 3.**
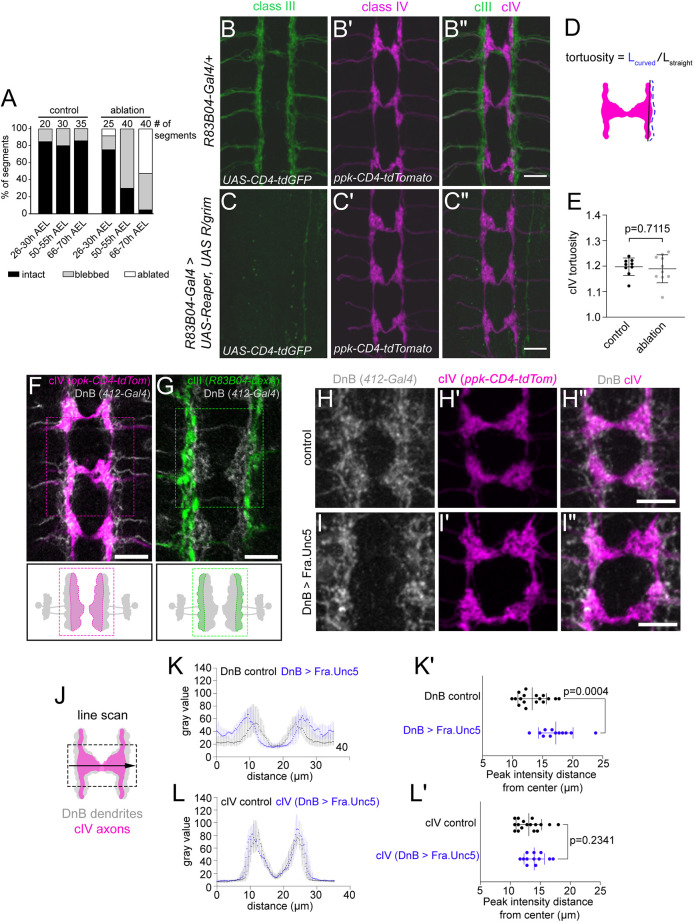
**cIV axon targeting is not altered after cIII neuron ablation or upon manipulation of a downstream target.** (A) Quantification of cIII axon loss at various stages of development. CNS segments A2-A6 were analyzed individually and pooled by larval age and genotype. Numbers of segments analyzed per group are noted above bars. (B-B″) Maximum-intensity projections of control cIII (B) and class IV (cIV) (B′) axons labeled by *R83B04-Gal4* and *ppk-CD4-tdTomato*, respectively. (C-C″) Maximum-intensity projections of cIII (C) and cIV (C′) axons upon ablation of cIII neurons with *R83B04-Gal4*. (D) Schematic of tortuosity analysis. Tortuosity was calculated using the arc-chord method. (E) cIV tortuosity in control larvae and larvae with cIII neurons ablated. Each dot represents one larva, and means±s.d. are shown. Control, *n*=9; ablation, *n*=10. *P*=0.7115 by two-tailed unpaired *t*-test. (F,G) Single-plane (*z*=1 µm) images of cIV axons (F) and cIII axons (G) co-labeled with down-and-back (DnB) dendrites. Drawings below depict one pair of bilateral DnB neurons with cIV (nociceptive) recipient dendritic region in magenta and cIII (touch) recipient dendritic region in green. (H-I″) Maximum-intensity projections of DnB dendrites (labeled by *412-Gal4,UAS-CD8-GFP*) and cIV terminals (labeled by *ppk-CD4-tdTomato*) from two CNS abdominal segments in control (H-H″) and DnB-mistargeted (I-I″) larvae. DnB dendrites were mistargeted by forced expression of a chimeric Fra.Unc5 protein, which functions as a repulsive Netrin receptor (Keleman and Dickson, 2001). Note that cIV axon scaffold patterning is not noticeably disrupted. (J) Schematic of line scan analysis used for quantification of DnB dendrite and cIV axon patterning upon DnB dendrite mistargeting. Quantification method is detailed in the Materials and Methods. (K-L′) Quantification of DnB dendrite and cIV axon patterning upon DnB dendrite mistargeting with Fra.Unc5. K and L are pixel intensity plots showing fluorescence gray value at each point sampled along line scans of DnB dendrites and cIV axons. Note that DnB dendrites expressing Fra.Unc5 shift laterally compared with control (K), whereas no shift is observed for cIV axons upon DnB mistargeting (L). Plots in K and L show medians±interquartile ranges. K′ and L′ show quantification of peak pixel intensity distance from center. Each dot in K′ and L′ represents one larva, and means±s.d. are shown. *P*-values were determined by two-tailed unpaired *t*-test. DnB (control) versus DnB (DnB>Fra.Unc5), *P*=0.0004; cIV (control) versus cIV (DnB>Fra.Unc5), *P*=0.2341. Control, *n*=17 larvae; DnB>Fra.Unc5, *n*=12 larvae. Pixel intensity plot data in K and L are the same as in Fig. S2. Plots are arranged and color coded to directly compare cIV and DnB by genotype. Larvae quantified in A were imaged live. All other samples shown in images were labeled using immunohistochemistry. Scale bars: 10 µm.

We next asked whether cIII axons are reciprocally required for cIV axon targeting by examining the cIV axon scaffold after cIII ablation. Targeted expression of Reaper and a chimeric Reaper/Grim protein (Wing et al., 2001) under the control of *R83B04-Gal4* resulted in cIII axon blebbing or entirely missing axons in 24% of segments in first-instar larvae (26-30 h AEL), 70% of segments in second-instar larvae (50-55 h AEL) and 95% of segments in larvae aged 66-70 h AEL (Fig. 3A). In third-instar larvae, we did not observe obvious changes in the cIV axon scaffold, such as axon sprouting or misrouting, in cIII-ablated larvae compared with controls (Fig. 3B-C″). We also examined whether elimination of cIII axons resulted in an irregular lateral cIV axon boundary by measuring the tortuosity of the lateral edge of the cIV axon scaffold in control and cIII-ablated larvae (Fig. 3D,E). We found no significant difference in cIV tortuosity between control and cIII-ablated larvae (Fig. 3E). Given that the ablations removed cIII axons after targeting, these results suggest that factors other than cIII axons are responsible for maintaining cIV axon patterning in third-instar larvae. Because we could not achieve earlier ablation, our results do not rule out an early role for cIII axons in impacting cIV axon patterning.

We also examined whether a synaptic target of cIV and cIII neurons, the DnB neurons (Burgos et al., 2018), might influence cIV targeting. Consistent with our previous study (Burgos et al., 2018), co-labeling either class of sensory neuron with DnBs showed that sensory axon terminals overlap with DnB dendrites but terminate on different dendritic regions (Fig. 3F,G). We induced dendrite mistargeting in DnB neurons by expressing a chimeric Netrin receptor, *UAS-fra.unc5* (Keleman and Dickson, 2001), in DnB neurons and asked whether cIV axons responded by changing their position. The Fra.Unc5 chimera behaves as a repulsive Netrin receptor and prevents commissural axons from crossing the midline (Keleman and Dickson, 2001). Fra.Unc5 expression in DnB neurons led to a clearing of their midline dendrites as measured by the distance from the midline of peak fluorescence intensity (Fig. 3H,I,J-K′, Fig. S2); however, we did not detect changes in cIV axons using the same quantification (Fig. 3H′,I′,J,L,L′, Fig. S2). These results suggest that cIV axon and DnB dendrite targeting can be decoupled, and subcellular cIV axon targeting is unlikely to be driven primarily by cues from DnB dendrites.

### cIV axons affect the distribution of cIII neuron presynaptic sites

**Fig. 4.**
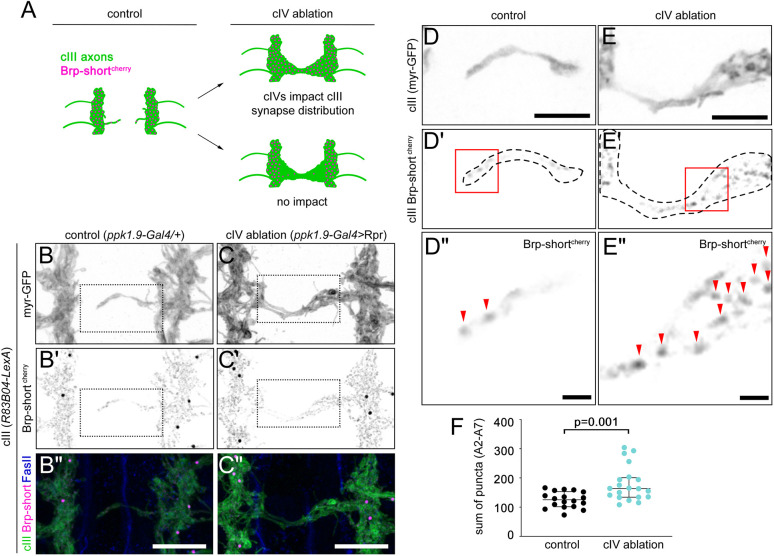
**Mistargeted cIII axon terminals accumulate a presynaptic marker.** (A) Potential outcomes and interpretations of cIV ablation on cIII presynapses. (B-C″) cIII axons from CNS segment A4 labeled by myr-GFP and presynaptic sites in cIII neurons labeled by Brp-short^cherry^ in control (B-B″) and cIV-ablated (C-C″) larvae. Brp-short^cherry^ channels in B′-C″ and D′-E″ were masked by the corresponding GFP channel, and a background subtraction was applied using a custom ImageJ macro (see Materials and Methods). Unmasked images without background subtraction are in Fig. S3. (D-E′) Single-plane images (*z*=0.5 µm) of commissural branches from B, B′, C and C′, respectively. (D″,E″) Enlarged images of the boxed regions in D′ and E′, respectively. Arrowheads point to Brp-short^cherry^ puncta. (F) Sum of Brp-short^cherry^ puncta in medial region from CNS segments A2-A7. *P*=0.001 by two-tailed Mann–Whitney test. Control, *n*=18 larvae; ablation, *n*=20 larvae. Each dot represents one larva, and medians±interquartile ranges are shown. All samples shown in images were labeled using immunohistochemistry. Scale bars: 10 µm (B″,C″); 5 µm (D,E); 1 µm (D″,E″).

Our finding that cIII axons mistarget in the absence of cIV neurons prompted us to ask whether cIV axons influence the distribution of cIII neuron presynaptic sites. To investigate synapse localization upon cIV ablation, we labeled cIII axons and presynapses with membrane-targeted GFP and Brp-short^cherry^ (Berger-Muller et al., 2013), respectively. We predicted that if cIV axons prevent cIII axons from synapsing in the nociceptive neuropil, cIV ablation should lead to an increase in Brp-short^cherry^ sites in this region (Fig. 4A). In control larvae, we observed Brp-short^cherry^ puncta in the longitudinal processes of cIII axons and in the occasional commissural branches that extended towards the midline (Fig. 4B-B″,D-D″, Fig. S3A-A″). In cIV-ablated larvae, we found a significant increase in puncta in the midline region compared with those in controls (Fig. 4C-C″,E-F, Fig. S3B-B″), indicating that the presence of cIV neurons limits the number of presynapses formed by cIII axon terminals within the nociceptive neuropil. cIV neurons could limit cIII presynapse distribution either by directly inhibiting cIII synapse formation or by preventing extensive midline growth of cIII axons. To distinguish between these models, we normalized the number of Brp-short^cherry^ puncta by the volume of cIII axons in the midline region (Fig. S3C). When normalized to cIII axon volume, no significant difference in puncta density was observed between control and cIV-ablated groups (Fig. S3C). These results suggest that cIV axons impact cIII connectivity by restricting their axons to the lateral region of the neuropil, outside of the nociceptive region, which restricts synapse formation to this same region. We propose that this effect of cIV neurons on cIII axons underlies the segregation of touch and nociceptive input onto central targets.

### cIV neuron ablation alters the location of putative cIII inputs onto a nociceptive interneuron

**Fig. 5.**
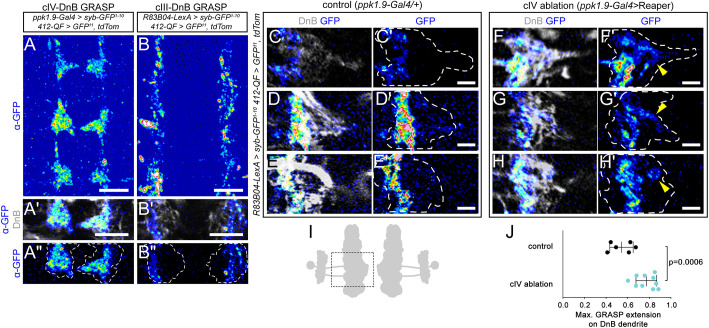
**cIV ablation alters the location of putative cIII synapses with DnB nociceptive interneurons.** (A-B″) Anti-GFP antibody labeling of reconstituted GFP (pseudocolored) between cIV and DnB neurons (A-A″) and cIII and DnB neurons (B-B″). GFP reconstitution across synaptic partners (GRASP) co-labeled with DnB marker (A′,B′) shows that cIV-DnB GRASP signal is seen in the medial region of the DnB dendritic arbor, whereas cIII-DnB GRASP is restricted to the lateral margin of the DnB dendrites. Dashed lines in A″ and B″ indicate DnB dendritic territory. A and B are maximum-intensity projections; A′, A″, B′ and B″ are single-plane (*z*=0.5 µm) images. (C-H′) Examples of reconstituted GFP native signal between cIII axons and DnB dendrites in control (C-E′) and cIV-ablated (F-H′) larval VNCs. C, D, E, F, G and H show reconstituted GFP (pseudocolored) co-labeled with DnB dendritic marker (pseudocolored in gray). C′, D′, E′, F′, G′ and H′ show GFP channel (pseudocolored) with dashed outlines around DnB dendritic field. Reconstituted GFP in control CNS is restricted to lateral edge of DnB dendrite but extends medially when cIV axons are ablated, indicated by arrowheads. Images shown are single plane (*z*=1 µm). A rolling ball background subtraction (ImageJ) was applied equally to all images in C-H′ (see Materials and Methods). (I) Drawing of DnB neurons with box indicating region represented in images in C-H′. (J) Quantification of GRASP extension towards midline with respect to DnB dendrites. Each dot is one larva, and means±s.d. are shown. *P*=0.0006 by two-tailed unpaired *t*-test. Control, *n*=6 larvae; ablation, *n*=10 larvae. Scale bars: 10 µm (A-B′); 5 µm (C′,D′,E′,F′,G′,H′).

Given the above results, we hypothesized that touch- and nociceptive-specific patterns of connectivity with DnB neurons might be determined by their distinct axon placements rather than a localized cell surface code. To investigate this, we used synaptic GFP reconstitution across synaptic partners (GRASP) (Macpherson et al., 2015) to detect putative cIII and cIV connectivity with DnB neurons. GRASP labeling for each class reflected their respective axon projection pattern: cIV-DnB GRASP was concentrated near the midline, whereas cIII-DnB GRASP formed parallel longitudinal tracts (Fig. 5A,B). We introduced a DnB marker, *412-QF>QUAS-mtdTomato*, to examine GRASP with respect to DnB dendrites. cIV-DnB GRASP was detected throughout DnB dendrites except for a narrow lateral margin, whereas cIII-DnB GRASP was restricted to the lateral part of the dendritic field (Fig. 5A′-B″). Thus, class-specific targeting of touch and nociceptive axon terminals are reflected in modality-specific patterns of subcellular connectivity with DnB neurons.

We next tested the role of axon interactions in specifying the location of inputs onto DnB dendrites. We ablated cIV neurons and simultaneously performed GRASP to assess connectivity between cIII and DnB neurons. We again introduced a DnB dendritic marker (mtdTomato) to examine GRASP distribution on the DnB dendritic field. We focused on the DnB dendritic regions near the midline that receive input from cIV branches (Fig. 5C-J). In control larvae, cIII-DnB GRASP was restricted to the lateral region of DnB dendrites (Fig. 5C-E′). When cIV neurons were ablated, GRASP was observed along the lateral region of DnB dendrites, but, unlike in controls, GRASP frequently extended towards the midline (Fig. 5F-H′). We measured the maximum distance that GRASP signal extended along the DnB dendrite and found a significant increase in cIV-ablated larvae compared with that in controls (Fig. 5J). This result suggests that cIV axons restrict the connectivity of cIII axons to the lateral ‘touch’-associated region of DnB dendrites. Comparison of relatively restricted GRASP signal to the extensive midline labeling with Brp-short^cherry^ (Fig. 4E-E″) suggests that other targets besides DnBs receive ectopic input from cIII neurons when cIV neurons are ablated.

### Live imaging of early somatosensory axon development reveals successive targeting of cIII and cIV axons

**Fig. 6.**
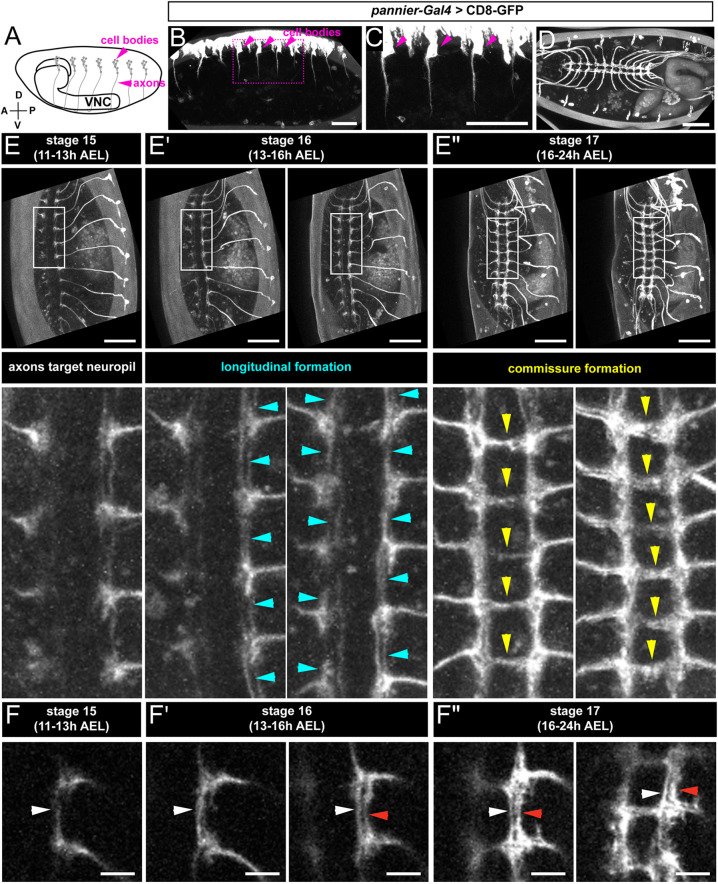
**Development of a live-imaging platform for somatosensory axons during early embryogenesis.** (A) Schematic of embryonic dorsal cluster sensory neurons and axon projections. (B-D) Maximum-intensity projections of *pnr-Gal4>UAS-CD8-GFP* expression in embryo. (B) Lateral view of stage 14/15 embryo. (C) Enlarged view of the boxed area in B, showing axon outgrowth from the dorsal cell cluster (indicated by magenta arrowheads). (D) Ventral view of stage 17 embryo. Note that *pnr-gal4* does not label CNS neurons. (E-E″) Time-lapse imaging of sensory axon targeting with *pnr-gal4*. (Top row) Maximum-intensity projections of the embryo at stages 15 (E), 16 (E′) and 17 (E″) corresponding to 11-13 h AEL, 13-16 h AEL and 16-24 h AEL at 25°C, respectively. (Bottom row) Enlarged views of boxed regions in the top row. Images were taken from the ventral side of the embryo. Anterior is up and posterior is down. (E) Sensory axon terminals arborizing within corresponding neuromeres. (E′) Embryo at two phases in stage 16. Left images (top and bottom) show early phase, during which longitudinal projections are formed. Right images (top and bottom) show later phase, during which projections thicken to form longitudinal connectives. Cyan arrowheads indicate projections forming longitudinal connectives. (E″) Embryo at two points during stage 17. Note that commissural connectives, indicated by yellow arrowheads, have formed. (F-F″) Single slice images of A6 and A7 taken from embryo in E-E″ at the same time points. (F) Neuromeres A6 and A7 from one side of stage 15 embryo. White arrowhead indicates axon process forming longitudinal connective. (F′) Images from stage 16, during which a second longitudinal connective (red arrowhead) forms immediately lateral to the first bundle (white arrowheads). Note that both bundles of processes remain separated. (F″) Two points during stage 17 as longitudinal bundles thicken and commissural connectives form. Note that commissural axons extend from the medially positioned layer (white arrowheads) and the layers remain segregated. Scale bars: 50 µm (B-E″); 10 µm (F-F″).

To investigate the origin of class-specific axon segregation, we developed a method for live imaging of somatosensory axons and their terminal processes. Mediolateral segregation of da neuron axons can be seen as early as embryonic stage 17 (16-24 h AEL) (Grueber et al., 2007). Monitoring of axon targeting therefore required reporters that are expressed early in multiple classes of da neurons with little or no expression in central neurons. Such reporters have not previously been described, but we hypothesized that reporters of early patterning genes might fulfill these requirements because they are expressed in restricted domains very early during embryonic development. One such early patterning gene is *pannier* (*pnr*), a GATA transcription factor involved in dorsal cell fate determination (Calleja et al., 2000; Heitzler et al., 1996; Ramain et al., 1993). An enhancer trap inserted in the *pnr* locus (*pnr-Gal4*) drives expression in embryonic dorsal ectoderm (Calleja et al., 1996; Stronach et al., 2014). We found that *pnr-Gal4* also drives *20XUAS-mCD8-GFP* expression in dorsal cluster sensory neurons, including ddaC (cIV) and ddaF (cIII) (Fig. S4A). Dorsal sensory axons could be visualized during their extension and throughout embryonic development (Fig. 6A-D). Importantly, *pnr-Gal4* did not drive reporter expression in central neurons, which might otherwise obscure sensory axon terminals (Fig. 6D).

Live imaging of *pnr-Gal4; 20XUAS-mCD8-GFP* embryos revealed that sensory axon bundles from each abdominal segment entered the VNC at approximately stage 15 (corresponding to 11-13 h AEL at 25°C) (Fig. 6E) and initially arborized within their corresponding neuromere before extending anteriorly and posteriorly to form longitudinal connectives (Fig. 6E′). Commissural extensions were first observed at stage 16 (13-16 h AEL) (Fig. 6E′), and, by stage 17, midline crossing was observed in all segments (Fig. 6E″). Because all bundled axons were labeled by GFP, we were not able to identify individual axons, but we can infer that the substantial midline crossing arose from axons of cIV neurons. In four embryos, we observed sequential generation of longitudinal connectives in a medial-to-lateral sequence. A medial longitudinal branch arose first (white arrowheads in Fig. 6F-F″ and Fig. S4B) from the same axon bundle that produced the midline extension, followed by distinct, more lateral, longitudinal bundle (red arrowheads in Fig. 6F′,F″ and Fig. S4B). These results are consistent with axon segregation via sequential targeting of cIV and cIII axons.

## DISCUSSION

Neuronal dendrites often receive inputs from multiple distinct cell types across their arbor. These inputs can segregate into discrete domains that reflect functional features of the inputs (Spruston, 2008). How such segregation arises during development is still poorly understood. We studied this problem in a relatively simple somatosensory circuit in *Drosophila* in which axons of cIII gentle touch and cIV nociceptive neurons terminate in adjacent neuropil regions and synapse onto distinct regions of downstream DnB dendrites. We found that touch receptor axons are competent to grow and synapse more broadly in the neuropil and DnB dendritic field but are restricted from doing so by the presence of nociceptive axons. Live-imaging data support a model in which distinct arborization times of axon cohorts contribute to modality-specific targeting and subcellular specificity of somatosensory axons.

### A role for axon interactions in somatosensory circuit wiring

By disrupting normal patterns of sensory axon targeting and ablating cohorts of sensory neurons, we discovered a role for heterotypic cell interactions in modality-specific wiring of somatosensory circuitry. Ablation of cIV nociceptive neurons resulted in extension of cIII touch receptor axons into the nociceptive recipient neuropil. Notably, our data suggest that ablation occurred after initial axon terminal targeting, supporting a conclusion that axon interactions are required to maintain the boundary between cohorts. We suspect that this ongoing maintenance requirement arises because axons expand connectivity throughout larval development in a growing neuropil (Gerhard et al., 2017), which involves ongoing plasticity and growth. A method for earlier ablation of nociceptors could be used to test roles for axon interactions during initial targeting.

Our analysis of midline crossing by cIII axons also revealed that crossing occurs more frequently in anterior segments than in posterior segments (Fig. 1J′) in control larvae and cIV-ablated larvae. Although we do not know the basis for this result, we speculate that it could reflect spatial or temporal differences in axon dynamics along the anterior–posterior (A-P) axis during development. In larvae with cIV axons ablated, cIII midline crossing was increased in segments A2-A7 but following a similar trend in which anterior segments are crossed more than posterior segments, suggesting that similar factors contribute to A-P differences in midline crossing in control and cIV-ablated larvae.

Whereas cIII–cIV interactions seem to be required for proper cIII axon targeting, ablating cIII neurons did not cause detectable changes in cIV axon patterning. Thus, axon interactions appear to influence targeting in a unidirectional manner. Our live-imaging data are consistent with sequential targeting of axons to medial and then lateral neuropil. Differential timing could explain the unidirectional influence of axon–axon interactions if the first-arriving axons influence later-arriving axons but not vice versa. A weakness of our live-imaging data is the lack of differential labeling of cIII and cIV axons. Future live imaging with tools that enable early, class-specific labeling could further test our model. In mice, DiI labeling of sensory afferents at various stages of development revealed that different classes of neurons project to the dorsal horn sequentially based on final position (Ozaki and Snider, 1997), suggesting a possible parallel between invertebrate and vertebrate somatosensory circuit formation. Our results are also consistent with findings of sequential targeting in olfactory map formation (Sweeney et al., 2007; Takeuchi et al., 2010) and the *Drosophila* ellipsoid body (Xie et al., 2017). In olfactory map formation, early-arriving axons repel late-arriving axons through Semaphorin signaling. Thus, an important future direction is to examine roles for axon guidance ligands and receptors in somatosensory axon segregation.

Homotypic interactions may also contribute to modality-specific wiring. Both cIII and cIV axons appear to associate with other axons of the same class. A combination of homotypic adhesive forces within cIII and cIV axon cohorts, and repulsion between cohorts could drive modality-specific axon convergence, as is the case for mouse olfactory sensory neurons (Serizawa et al., 2006). Axon pre-target sorting could conceivably contribute to segregation in target zones (Cioni et al., 2018; Sitko et al., 2018). In the mouse optic tract, retinal ganglion cell (RGC) axons that project ipsilaterally and contralaterally are segregated, with ipsilateral projections situated laterally (Sitko et al., 2018). Such pre-target sorting appears to arise from differential adhesion within RGC axon cohorts. Examining somatosensory axon organization within the pathways that deliver axons to their targets in the CNS could shed light on whether pre-target sorting is observed in this system and possible roles in somatosensory circuit development.

### cIV nociceptors impact modality-specific subcellular wiring of cIII touch axons

cIII and cIV axons target complementary territories of DnB dendrites, forming touch- and nociceptive-specific patterns of connectivity (Burgos et al., 2018). Our data indicate that these patterns are influenced by the relative placement of axon terminals, which, in turn, is mediated by a heterotypic axon–axon interaction. cIV ablation experiments revealed that the restriction of cIII axons to the ‘touch’ synaptic zone is dependent on the presence of neighboring cIV axons. This result reveals that axon–axon interactions contribute to subcellular specificity of cIII axon wiring. Several interneuron classes are postsynaptic to cIV neurons (Burgos et al., 2018; Gerhard et al., 2017; Hu et al., 2017; Kaneko et al., 2017; Ohyama et al., 2015; Takagi et al., 2017; Yoshino et al., 2017), including at least four classes that are also postsynaptic to adjacent cIII axons (Burgos et al., 2018; Jovanic et al., 2019; Takagi et al., 2017). An intriguing question is whether axon–axon interactions coordinate modality-specific subcellular wiring of cIII and cIV axons with multiple cohorts of shared interneuron targets. Notably, an adjacent layer of cII somatosensory axon terminals is positioned immediately lateral to the cIII axons (Grueber et al., 2007), raising the interesting possibility that successive neighbor axon–axon interactions determine somatosensory synaptic organization more generally. Lastly, whether axon–axon interactions play a role in partner selection in the somatosensory system in addition to subcellular synaptic specificity is not known and will be an important question for future studies.

Some of the features of somatosensory axon targeting are reminiscent of the mechanisms that contribute to connectivity in the vertebrate visual system. Synapses of T6 and T7 bipolar cells (BCs) converge on ON-sustained retinal ganglion cells (A_ON-S_ RGCs). Determination of the relative abundance of inputs depends on a number of cell-autonomous and cell non-autonomous mechanisms (Okawa et al., 2014), including interactions between major inputs (T6 BCs) and minor inputs (T7 BCs). When the T6 BCs are ablated, T7 BC input is expanded, indicating that growth-inhibiting axon–axon interactions between these two different cell types normally define the relative abundance of synaptic inputs. Ablation of T6 BCs also resulted in A_ON-S_ RGC dendrite mistargeting and formation of ectopic synapses. An additional focus of future studies will be to examine whether cIV ablation affects DnB dendritic arborization and, if so, impacts on connectivity with cIII axons and potentially partner selection. Identification of an analogous mechanism in the *Drosophila* somatosensory circuit and implication of axon arrival time in the establishment of the major and minor inputs opens this phenomenon to genetic analysis that could inform understanding of other systems.

## MATERIALS AND METHODS

### Fly strains and maintenance

*Drosophila melanogaster* were reared using standard methods. Experimental crosses were maintained at 25°C. Third-instar larvae were used unless otherwise specified. For most experiments, males and females were used. For some genetic ablations, males were the control genotype and females were the experimental genotype because a *UAS-reaper* transgene inserted on the X chromosome was used. Genotypes for each figure are listed in Table S1. The following lines were used: *ppk-CD4-tdTomato* (II) (Han et al., 2011); *R83B04-Gal4* (Jenett et al., 2012); *ppk1.9-Gal4* (Ainsley et al., 2003); *ppk-Gal4* (Grueber et al., 2007); *UAS-CD8-Cherry*; *13XLexAop2-mCD8-GFP* (BL32205; Pfeiffer et al., 2010); *R83B04-LexA* (this study); *UAS-Robo3* (Rajagopalan et al., 2000); *UAS-Reaper* (X) (McNabb et al., 1997); *412-QF* (Burgos et al., 2018); *QUAS-mtdTomato-3xHA* (Potter et al., 2010); *UAS-syb-GFP^1-10^* (Macpherson et al., 2015); *QUAS-CD4-GFP^11^* (a gift from the Sehgal laboratory; Cavanaugh et al., 2014); *LexAop-syb-GFP^1-10^* (Macpherson et al., 2015); *13XLexAop2-IVS-myr-GFP* (BL32210; a gift from the Mann laboratory, Columbia University, NY, USA); *ppk*-*CD4-tdGFP* (III) (Han et al., 2011); *8XLexAop-Brp-short^cherry^* (Berger-Muller et al., 2013); *13XLexAop2-IVS-myr-GFP* (BL32212; Pfeiffer et al., personal communication to FlyBase, FBrf0212441); *13XLexAop2-6XmCherry* (Shearin et al., 2014); *412-Gal4* (Burgos et al., 2018); *UAS-mCD8-GFP* (Lee and Luo, 1999); *UAS-fra.unc5* (Keleman and Dickson, 2001); *UAS-reaper* (II) (BL5824; Aplin and Kaufman, 1997); *UAS-reaper/grim* (a gift from John Nambu; Wing et al., 2001); *pnr-Gal4* (BL3039; Calleja et al., 1996); *20XUAS-mCD8-GFP* (II and III); *UAS-CD4-tdGFP* (III) (Han et al., 2011).

### Immunohistochemistry

Larvae were dissected in 1× PBS, fixed in 4% paraformaldehyde (Electron Microscopy Sciences) in 1× PBS for 15 min, rinsed for 15 min in 1× PBS+0.3% Triton X-100 (Sigma-Aldrich) (PBS-TX) and blocked for at least 1 h in 5% normal donkey serum (Jackson ImmunoResearch) at 4°C. Larvae were incubated overnight in primary antibodies at 4°C, washed for at least 60 min in PBS-TX, and incubated for either 1-2 days at 4°C or 2 h at room temperature in species-specific, fluorophore-conjugated secondary antibodies. For Brp-short^cherry^ labeling, antibody incubations were done in 5% normal donkey serum to minimize non-specific binding. Tissue was mounted on poly-L-lysine-coated coverslips, dehydrated in ethanol series, cleared in xylenes and mounted in DPX (Electron Microscopy Sciences).

Embryos were collected on grape plates and aged at 25°C. Embryos were dechorionated for 2 min in 100% bleach, rinsed with embryo wash solution (7% NaCl, 0.5% Triton X-100) diluted 1:10, then with H_2_O. Dechorionated embryos were fixed in a 50/50 mixture of heptanes and 4% paraformaldehyde for 20 min, washed several times in 100% methanol, washed in PBX-TX for 90 min, and blocked in 5% NDS for 1 h at 4°C. Embryos were incubated overnight in primary antibodies at 4°C, washed in PBS-TX and incubated overnight at 4°C in species-specific, fluorophore conjugated secondary antibodies. Gut morphology was used to determine embryo stage for imaging.

Primary antibodies used were chicken anti-GFP (1:1000; Abcam, ab13970), rabbit anti-DsRed (1:200-1:500; Clontech, 632496), mouse anti-Fasciclin II (FasII) (1:10; Developmental Studies Hybridoma Bank, 1D4 anti-Fasciclin II), mouse anti-GFP (1:100; Sigma-Aldrich, G6539), mouse anti-Cut (1:20; Developmental Studies Hybridoma Bank, 2B10), goat anti-tdTomato (1:1000; LsBio, LS-C340696-600). Secondary antibodies used were from Jackson ImmunoResearch: Alexa Fluor 488 donkey anti-chicken (703-545-155), Alexa Fluor 647 donkey anti-mouse (715-605-150), Rhodamine Red-X donkey anti-rabbit (711-295-152), Alexa Fluor 488 donkey anti-mouse (715-545-150), Rhodamine Red-X donkey anti-goat, 705-295-147). All secondary antibodies were used at 1:200.

### GRASP

For native GRASP, third-instar larvae were dissected in PBS, fixed in 4% paraformaldehyde (Electron Microscopy Sciences) in PBS for 15 min, rinsed several times in PBS, mounted in Vectashield (Vector Labs) and imaged immediately. For antibody detection of GRASP using mouse anti-GFP antibody (Sigma-Aldrich, G6539), the above immunohistochemistry protocol was used.

### Generation of transgenes

The *R83B04* fragment was amplified from *w^1118^* genomic DNA using primer sequences provided by Janelia FlyLight [left, 5′-GAGGAGACCCCAATGAGTGCTAGTT-3′; right, 5′-GGCACGTATTTGAGGTGTTCTACGC-3′]. The left primer was modified with a 5′ cacc to enable directional cloning using a pENTR-D/TOPO cloning kit (Thermo Fisher Scientific) to generate *pENTR-R83B04*. The *R83B04* fragment was then transferred to *pBPLexA::p65Uw* (Addgene plasmid #26231) via Gateway LR Clonase recombination (Thermo Fisher Scientific) reaction to generate *pBPLexA::p65Uw-R83B04*. Transgenic lines were generated using PhiC31-mediated integration to attp2. Injections were performed by Best Gene Inc.

### Live imaging

#### cIV neuron ablations

Embryos were collected on grape plates and aged at 25°C. First- and second-instar larvae were placed in a drop of a mixture of Halocarbon oils 27 and 700 (Sigma-Aldrich), covered with a size #0 coverslip and imaged live.

#### cIII neuron ablations

Embryos were collected on grape plates and aged at 25°C. First- and second-instar larvae were placed in a drop of 100% glycerol, covered with a size #0 coverslip and imaged live.

#### Embryonic live imaging

Embryos were collected on grape plates and staged by gut morphology. Stage 14-16 embryos were manually dechorionated by rolling the embryo on double-sided tape. Dechorionated embryos were aligned ventral side up on premium microscope slides (Thermo Fisher Scientific) using embryo glue (made by dissolving Scotch double-sided tape in heptanes). Strips of double-sided tape and moistened (H_2_O) filter paper were placed on either side of the embryos to create a spacer between the slide and the coverslip and to provide moisture, respectively. The embryos were covered in a thin layer of Halocarbon 700 oil (Halocarbon Products Corp). A 24×50 mm 1.5H coverslip was placed on the chamber just before imaging. Time-lapse imaging was performed on a Zeiss 700 confocal microscope. Images were acquired every 17-30 min for 6-8 h.

### Image acquisition and processing

Images were acquired on Zeiss 510 Meta and Zeiss 700 confocal microscopes with LSM software (Zeiss) using a 40×/0.75 NA EC Plan-Neofluar objective. Image analysis was performed using ImageJ/Fiji (Schindelin et al., 2012; Schneider et al., 2012). Brightness/contrast were adjusted to visualize fine processes. A background subtraction (Rolling Ball, ImageJ) was applied to images in Fig. 3B-C″, Fig. 4B'-C″,D'-E″, Fig. 5C-H′ and was equally applied to control and experimental samples. The ImageJ plugin PoorMan3DReg was used to align some 3D images that were misaligned due to larval movement during live imaging. The PoorMan3DReg plugin was developed by Michael Liebling (University of California Santa Barbara, Santa Barbara, CA, USA) and uses a registration program based on Thevenaz et al. (1998). Large samples that required tiled *z*-stacks were stitched together in 3D using the Pairwise stitching ImageJ macro developed by Preibisch et al. (2009). Figures were made in Photoshop and Illustrator (Adobe).

### Quantification and statistical analysis

Files were blinded prior to quantification using a custom Python script (available at https://github.com/thomasbkahn/blinder_script). Statistical details for each experiment can be found in figure legends. *P*-values are shown in figures and listed in figure legends. Data were tested for normality using the Shapiro–Wilk test. For normally distributed data, two-tailed unpaired *t*-tests were used to compare two groups. For non-normal data, two-tailed Mann–Whitney *U*-tests were used to compare two groups and Kruskal–Wallis with Dunn's multiple comparisons test were used when comparing more than two groups. Statistical tests were performed in GraphPad Prism, MATLAB and Python. Sample size (*n*) refers to individual animals except where noted. No statistical methods were used to pre-determine sample size. Samples were excluded from analysis if the tissue was damaged or deformed during mounting, or if image quality was poor (low signal/high noise) such that the sample could not be analyzed. Animals were assigned to group based on genotype except where noted.

#### Quantification of midline crossing axons

CNS segments A2-A7 were scored. For each segment, axons were scored as crossing the midline if longitudinal axons were connected contralaterally by commissural axons and scored as non-crossing if no commissural axons were present or if commissural branches only extended partially.

#### Quantification of cIII axon volume in the midline region

CNS segments A2-A7 were analyzed. To differentiate between longitudinal and midline cIII axons in an unbiased manner, we limited the analysis to cIII axons that terminated medial to the ventral-ventromedial (VMv) FasII-positive tracts. The GFP channel (cIII membrane marker) was automatically thresholded using the Otsu method to create a binary neuron mask, and pixels due to noise were cleared manually. The ImageJ plugin Voxel Counter was used to measure total number of voxels in the binary neuron mask, and volume was calculated by multiplying by voxel dimensions. Five samples (four control, one ablation) could not be analyzed due to poor image quality (low signal/high noise) or tissue distortion during mounting, and were excluded from the dataset.

#### GRASP quantification

To select regions of interest (ROIs) for GRASP quantification (Fig. 5C-J), an ROI was drawn around DnB dendrites in a single *z*-plane. The ROI was overlaid onto the GRASP channel. The greatest length that GRASP signal extended from the lateral edge to the medial edge of the ROI (dendritic field) was measured. This was divided by the greatest lateral-to-medial length of the ROI, to adjust for differences in size of the dendritic field. Four to six ROIs were measured per CNS, and values were averaged to give one value per larva.

#### Quantification of Brp-short^cherry^ puncta

Images were acquired with a lateral pixel resolution of 0.1 µm and a *z*-step size of 0.5 µm. Images were processed prior to analysis using a custom ImageJ macro (available at https://github.com/lahammond/NeuronMask). Using this macro, the GFP channel (cIII membrane marker) was automatically thresholded using the Li method (Li and Tam, 1998) to create a binary neuron mask. This mask was applied to the Brp-short^cherry^ channel such that pixels outside of the neuron mask were cleared. Masking limited the analysis to pixels contained within cIII axons. A background subtraction was also applied to the Brp-short^cherry^ channel using a rolling ball radius of 3 pixels. To differentiate between longitudinal and midline cIII axons in an unbiased manner, we limited the analysis to cIII axons that terminated medial to the VMv FasII-positive tracts. Six ROIs were selected per CNS (neuromeres A2-A7) containing commissural cIII axon bundles. To quantify the number of Brp-short^cherry^ puncta, we used the FindFoci ImageJ plugin (Herbert et al., 2014), which uses machine learning to identify peak intensity regions in 3D images. Brp-short^cherry^ puncta were manually annotated for 38 ROIs (18 control and 20 ablation) and were used to train the FindFoci algorithm. The resulting algorithm had a precision value of 0.84, recall of 0.79 and F_1_ of 0.80. The algorithm was then applied to ROIs from all samples (*n*=108 control ROIs, *n*=120 ablation ROIs). For each larva analyzed, the number of Brp-short^cherry^ puncta from A2-A7 was summed to give one value per larva used for plotting and statistical analysis. Brp-short^cherry^ puncta density was calculated by dividing total number of puncta by total thresholded cIII axon volume (GFP channel) for each CNS.

#### Quantification of cIV scaffold tortuosity

Tortuosity was measured using the arc-chord ratio. A curved line (L_curved_) was manually drawn along the lateral edge of the cIV axon scaffold (left and right side for each CNS). A straight line was drawn to connect both ends (L_straight_). The arc-chord ratio was calculated (L_curved_/L_straight_) for each neuromere (A2-A6) and averaged to give one value per larva. A cIV scaffold that is completely straight should have tortuosity of 1, whereas a curved scaffold would have a tortuosity value >1.

#### Quantification of cIII and cIV axon ablation time course

CNS segments A2-A6 were analyzed individually and pooled by larval age and genotype. Segments were classified as intact (up to one bleb), blebbed (two or more blebs) or ablated (one or both hemisegments missing axons).

#### Quantification of cIV axon patterning upon DnB dendrite mistargeting

For each segment analyzed, a rectangular line scan (*x*=35 µm, *y*=20 µm, with a lateral pixel resolution of 0.29 µm) was applied to DnB (GFP) and cIV (RFP) channels centered at the midline. For each pixel along the *x*-axis, pixel intensity was vertically averaged (70 pixels, or 20 µm) using the Plot Profile analysis in ImageJ. The resulting plots (Fig. 3K,L, Fig. S2A-B″) show gray value at each point sampled along the 35 µm line scan to illustrate position, distribution and fluorescence intensity of DnB dendrites, cIV axons or both. CNS segments A2 and A4 were analyzed, and measurements were averaged for each larva. Statistical analysis was performed by comparing the peak fluorescence intensity distance from center between control and DnB mistargeted larvae, for both cIV axons and DnB dendrites.

## Supplementary Material

10.1242/develop.199832_sup1 Supplementary information
