# Supplementary material for: Axon–axon interactions determine modality-specific wiring and subcellular synaptic specificity in a somatosensory circuit
Source: Development. 2023 Mar 15;150(5):dev199832. doi: 10.1242/dev.199832 (PMC10112896; doi:10.1242/dev.199832)
Supplement: Supplementary information [file develop-150-199832-s1.pdf]

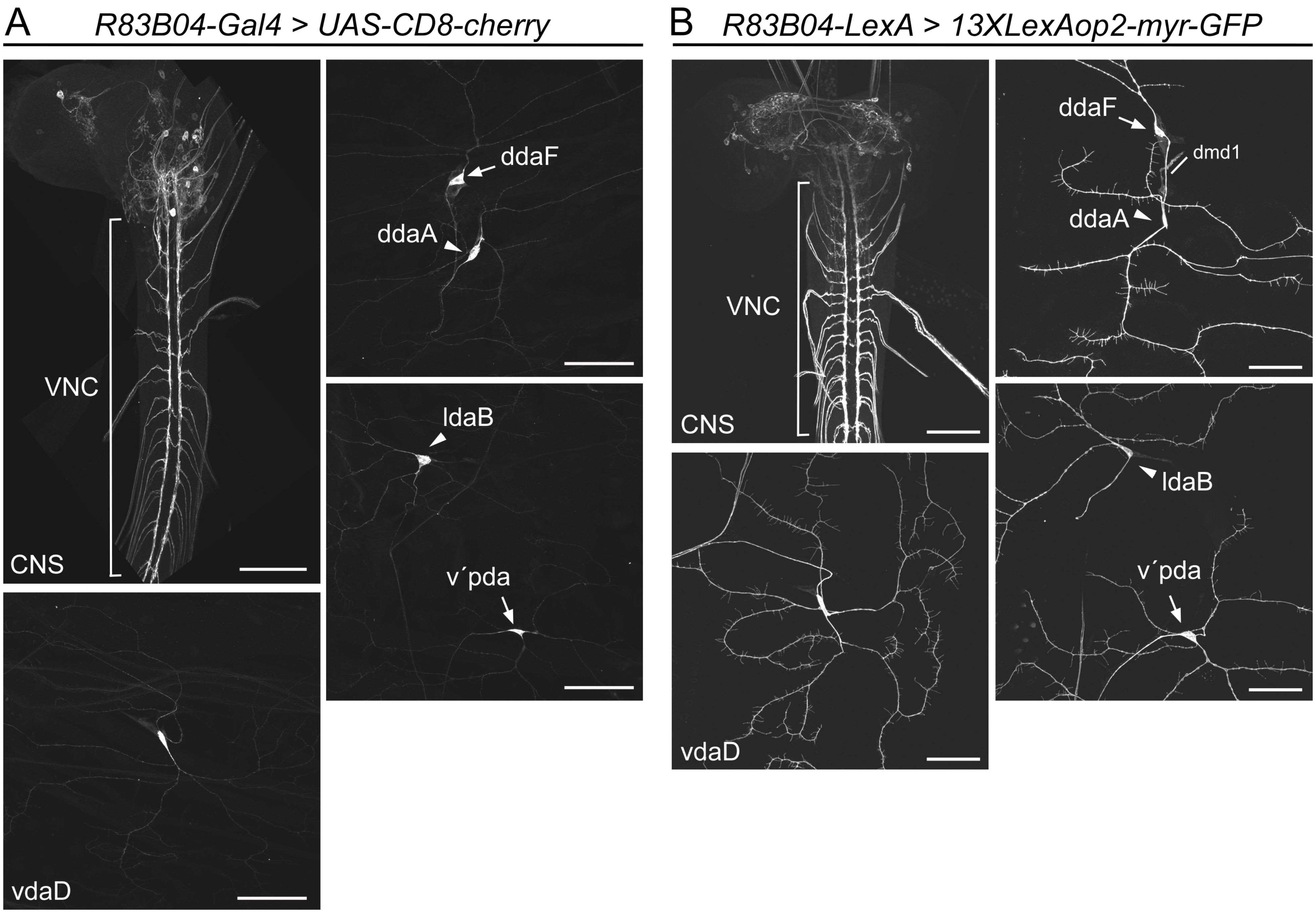

Fig. S1. ***RB3B04-Ga/4*** **and** ***RB3B04-LexA*** **are expressed in cIII da neurons.**

R83804-Ga/4 (A) and *R83804-LexA* (B) expression in third instar larval central nervous system (CNS) and body wall. All samples shown in images were labeled using immunohistochemistry . In CNS images, anterior is up and posterior is down. In body wall images (cell bodies, dendrites) dorsal is up and anterior is left. Scale bars = 50µm

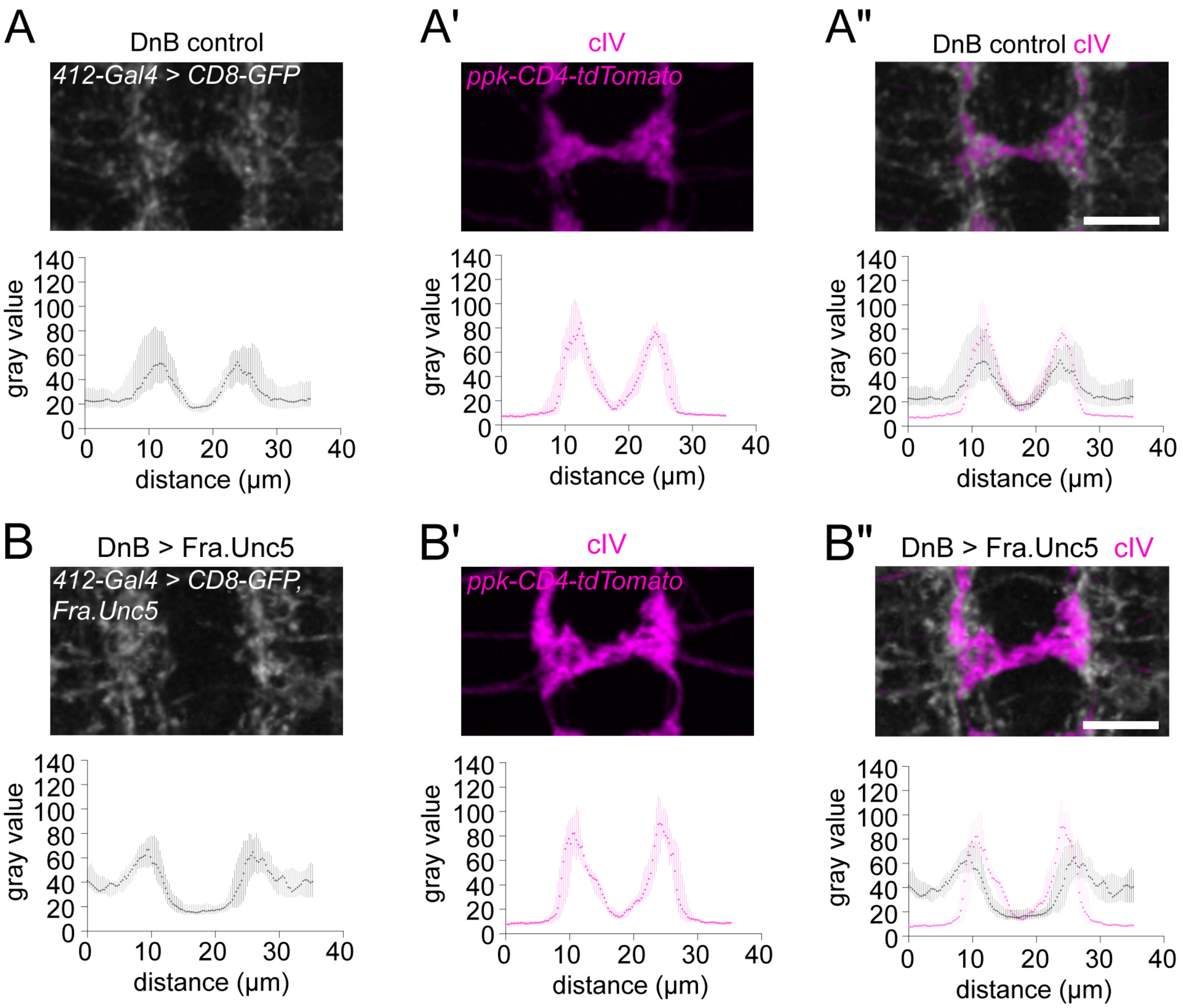

Fig. S2. Analysis of DnB dendrite and cIV axon patterning upon DnB dendrite mistargeting.

(A-B") Pixel intensity plots for control DnB dendrites and cIV axons (A-A") and DnB-mistargeted dendrites and cIV axons (B-B"). Plots show median ± interquartile ranges. n=17 larvae (control) n=12 larvae (Fra.Unc5). Representative images are included to illustrate phenotype and line scan placement. Pixel intensity plot data are the same as in Fig. 3K,L. Plots are arranged and color-coded here to illustrate patterning by neuron type. All samples were labeled using immunohistochemistry. Scale bars=1Oµm.

Development • Supplementary information

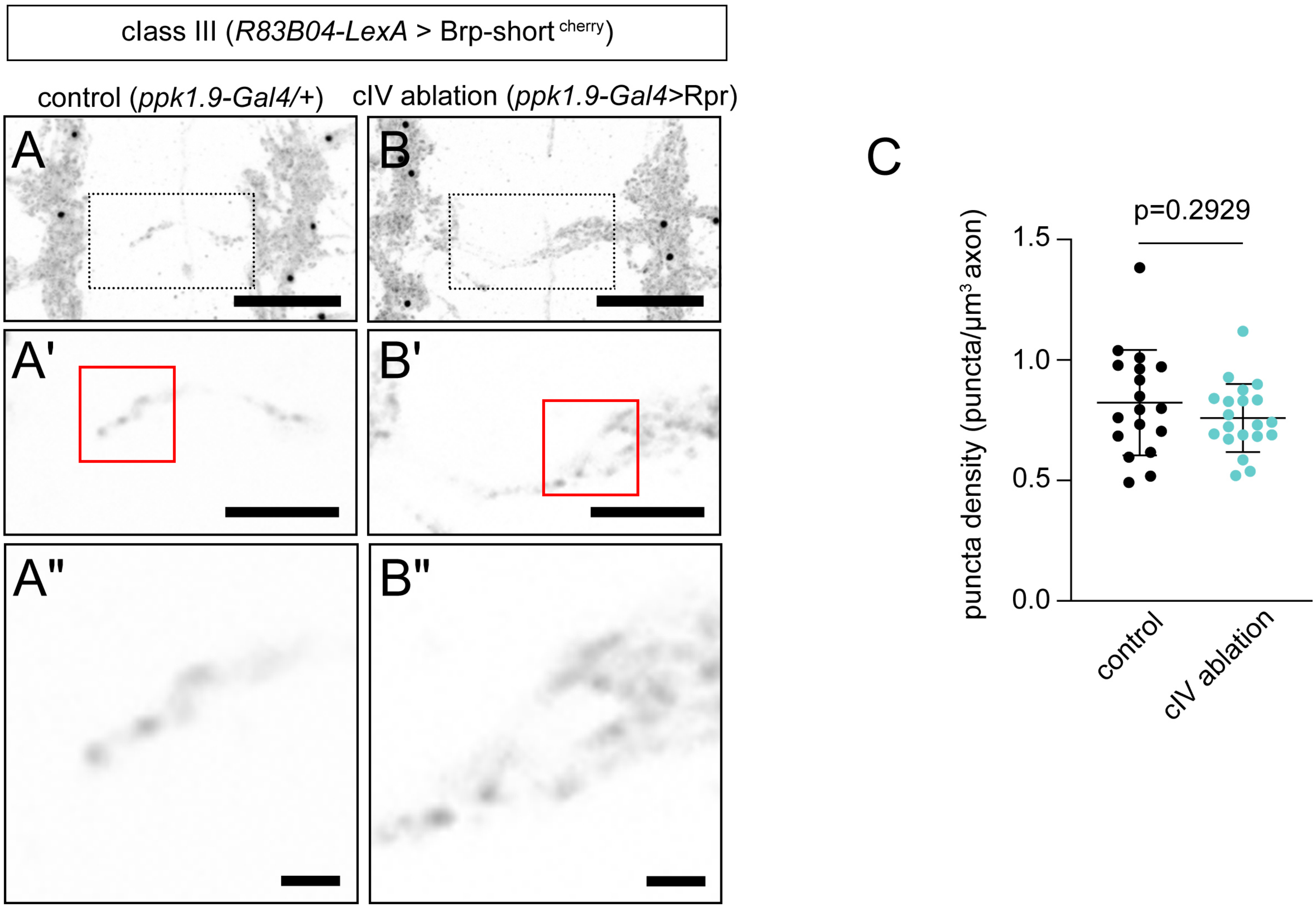

Fig. S3. Unmasked images of cIII presynapses labeled by Brp-short^{cherry} and puncta density analysis.

(A-A") Unmasked images of Brp-short^{cherry} labeling in control cIII axons corresponding to images in Figure 4. (A) is maximum intensity projection of axons in abdominal segment 4, (A') is single-plane (Z = 0.5µm) image of boxed region in A with colors inverted (A") is enlarged image of boxed region in A'.

(B-B") Unmasked images of Brp-short^{cherry} labeling in cIII axons after cIV ablation corresponding to images in Figure 4. (B) is maximum intensity projection of axons in abdominal segment 4, (B') is single-plane (Z = 0.5µm) image of boxed region in B with colors inverted, **(B")** is enlarged image of boxed region in B'.

(C) Quantification of Brp-short^{cherry} puncta density in control and cIV-ablated cIII axons. Puncta density for each larvae was calculated by dividing the total number of puncta by the total thresholded cIII axon volume for each CNS. control n=18, ablation n=20 larvae. Each dot represents 1 larva, and mean ± s.d. are shown. p=0.2929 by two-tailed t test.

Scale bars: (A,B) 1Oµm (A', B') 5µm (A", B") 1µm

Development • Supplementary information

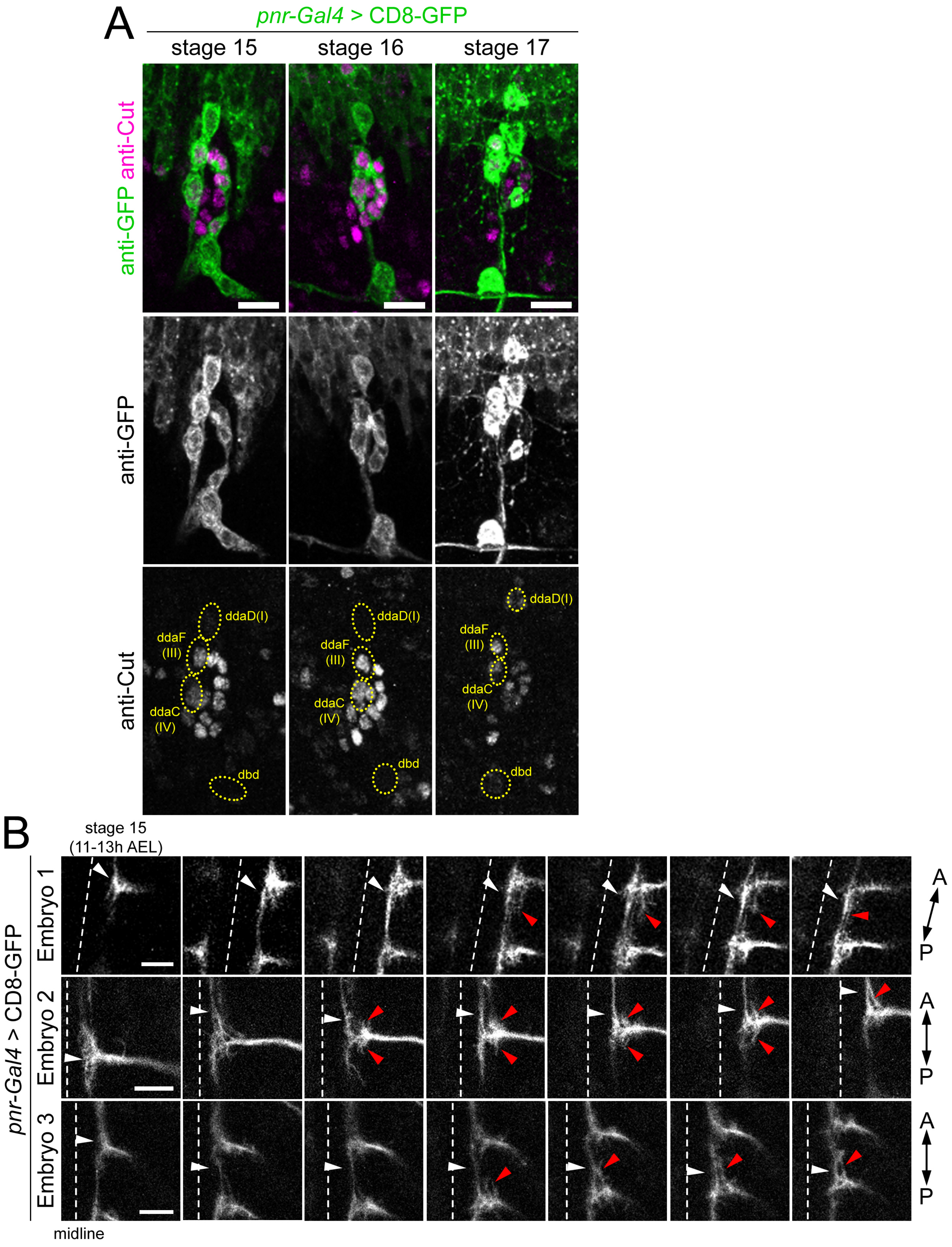

Development • Supplementary information

Fig. S4. Supplemental *pnr-ga/4* expression and embryonic live imaging.

(A) Co-labeling of *pnr-gal4>CD8-GF P* and Cut (anti-GFP and anti-Cut antibodies) in stage 15, 16 and 17 embryos. Of md neurons, *pnr-ga/4* labels ddaD (class I),ddaF (class 111), ddaC (class IV) and dbd. Cells were identified by soma position and Cut immunoreactivity (Grueber et al., 2003).

(B) Examples of media-lateral layer formation in three additional embryos. Dashed line indicates VNC midline,white arrowhead points to first layer formed, red arrowhead points to second layer formed. Note that a boundary is maintained between layers during formation. Images shown for Embryo 1 were acquired 54 minutes apart, images shown for Embryo 2 were acquired 30-60 minutes apart, images shown for Embryo 3 were acquired 46-69 minutes apart. Scale bars = 1Oµm

Development • Supplementary information

Table S1. Genotypes in figures

Figure 1
(B-B") *ppk1.9-Gal4, ppk-CD4-tdTomato; R83B04-LexA, 13XLexAop2-IVS-myr-GFP/+*
(C-C") *UAS-CD8-Cherry, 13XLexAop2-mCD8-GFP/+; ppk-Gal4, R83B04-LexA/+*
(D-D") *UAS-CD8-Cherry, 13XLexAop2-mCD8-GFP/UAS-Robo3; ppk-Gal4, R83B04-LexA/+*
(E-E", G) *Y/+; 13XLexAop2-IVS-myr-GFP/ppk1.9-Gal4, ppk-CD4-tdTomato; R83B04-LexA/+*
(F-F", H) *UAS-reaper/+; 13XLexAop2-IVS-myr-GFP/ppk1.9-Gal4, ppk-CD4-tdTomato; R83B04-LexA/+*
Figure 2
(B, D) +*; ppk1.9-Gal4/+; ppk-CD4-tdGFP/+*
(C, E) *UAS-reaper/+; ppk1.9-Gal4/+; ppk-CD4-tdGFP/+*
Figure 3
(B-B") *ppk-CD4-tdTomato/+; R83B04-Gal4, UAS-CD4-tdGFP/+*
(C-C") *ppk-CD4-tdTomato/UAS-reaper; R83B04-Gal4, UAS-CD4-tdGFP/UAS-R/grim*
(F) *ppk-CD4-tdTomato/+; 412-Gal4, UAS-mCD8-GFP/+*
(G) *13XLexAop2-6XmCherry/+; R83B04-LexA/412-Gal4, UAS-mCD8-GFP*
(H-H") *ppk-CD4-tdTomato/+; 412-Gal4, UAS-mCD8-GFP/+*
(I-I") *ppk-CD4-tdTomato/+; 412-Gal4, UAS-mCD8-GFP/UAS-fra.unc5*
Figure 4
(B-B", D-D") +*; ppk1.9-Gal4, 8XLexAop-Brp-short*^{cherry}*/+; R83B04-LexA, 13XLexAop2-IVS-myr-GFP/+*
(C-C", E-E") *UAS-reaper/+; ppk1.9-Gal4, 8XLexAop-Brp-short*^{cherry}*/+; R83B04-LexA, 13XLexAop2-IVS-myr-GFP/+*
Figure 5
(A-A") *ppk1.9-Gal4, QUAS-CD4-spGFP*^{11}*/+; 412-QF, QUAS-mtdTomato-3xHA/UAS-syb-GFP*$^{1-10}$
(B-B") *ppk1.9-Gal4, QUAS-CD4-spGFP*^{11}*/ LexAop-syb-GFP*$^{1-10}$*; 412-QF, QUAS-mtdTomato-3xHA/R83B04-LexA*
(C-E') *Y/+; ppk1.9-Gal4, QUAS-CD4-spGFP*^{11}*/ LexAop-syb-GFP*$^{1-10}$*; R83B04-LexA/412-QF, QUAS-mtdTomato-3xHA*
(F-H') *UAS-reaper/+; ppk1.9-Gal4, QUAS-CD4-spGFP*^{11}*/ LexAop-syb-GFP*$^{1-10}$*; R83B04-LexA/412-QF, QUAS-mtdTomato-3xHA*

Figure 6
(B-D) *20XUAS-mCD8-GFP; pnr-gal4, 20XUAS-mCD8-GFP/(TM6B)*<br>(E-F") *ppk-CD4-tdTomato; pnr-gal4, 20XUAS-mCD8-GFP/(TM6B)*
Supplemental Figure 1
(A) *UAS-CD8-Cherry, 13XLexAop2-mCD8-GFP/+; R83B04-Gal4/+*<br>(B) *13XlexAop2-IVS-myr-GFP/+; R83B04-LexA/(+ or TM6)*
Supplemental Figure 2
(A-A") *ppk-CD4-tdTomato/+; 412-Gal4, UAS-mCD8-GFP/+*<br>(B-B") *ppk-CD4-tdTomato/+; 412-Gal4, UAS-mCD8-GFP/UAS-fra.unc5*
Supplemental Figure 3
(A-A") +*; ppk1.9-Gal4, 8XLexAop-Brp-short^{cherry}/+; R83B04-LexA, 13XLexAop2-IVS-myr-GFP/+*<br>*(B-B*") *UAS-reaper/+; ppk1.9-Gal4, 8XLexAop-Brp-short^{cherry}/+; R83B04-LexA, 13XLexAop2-IVS-myr-GFP/+*
Supplemental Figure 4
(A-B) *20XUAS-mCD8-GFP; pnr-gal4, 20XUAS-mCD8-GFP/(TM6B)*

Supplementary Reference

Grueber, W. B., Jan, L. Y. and Jan, Y. N. (2003). Different levels of the homeodomain protein cut regulate distinct dendrite branching patterns of Drosophila multidendritic neurons. *Cell* **112**, 805-818.
